# An Experimental Approach to the Synthesis and Optimisation of a ‘Green’ Nanofibre

**DOI:** 10.3390/nano8060383

**Published:** 2018-05-30

**Authors:** Md. Nahid Pervez, George K. Stylios

**Affiliations:** Research Institute for Flexible Materials, School of Textiles and Design, Heriot-Watt University, Galashiels TD1 3HF, UK; nahid.tex92@gmail.com

**Keywords:** water-soluble chitosan (W_S_CHT), green nanofibre, ANOVA, S/N ratios

## Abstract

Currently, green-based materials are receiving attention in a quest to achieve a sustainable environment for human life. Herein, we report an investigation of developing a simple novel green nanofibre by using H_2_O_2_-assisted water soluble chitosan/polyvinyl alcohol (W_S_CHT/PVA) in the presence of water as an eco-friendly solvent. The effect of various process parameters on the mean fibre diameter was investigated based on the Taguchi L_9_ ($3^{4}$) orthogonal array experimental design. Optimal process parameters were determined using the signal-to-noise (S/N) ratio of diameter according to the ‘smaller-the-better’ concept. Accordingly, the smallest fibre diameter observed was 122 nm and it was yielded at solution concentration of 10%; a voltage of 16 kV; a flow rate of 0.7 mL h^−1^; and a collection distance of 8 cm. The implications of a green environmentally sustainable material impact on a number of diverse end uses.

## 1. Introduction

Green chemistry seeks to reduce or eliminate the generation and use of hazardous substances, and is built on a set of practices and principles in pursuit of that aim [1,2]. Industrial and academic research has, in recent years, shown increased interest in the fabrication of ‘green’ polymers from natural resources. More effort has gone into the replacement of polymers derived from petroleum with sustainable natural biopolymers that are biodegradable, environment-friendly, and require the consumption of less energy for their renewal [3,4]. Green electrospun materials may provide new end uses, such as protection against microorganisms by using solvents that are environmentally friendly, non-toxic, and low cost. Water-based solvents and materials provide a good example.

Electrospun nanofibres are generally made by electrospinning organic polymers [5], and possess high porosity, good stability, and a large surface area to volume ratio [6]. The end uses of electrospun nanofibres are wide-ranging, such as composites, catalysis, filters, tissue engineering, drug delivery, and wound healing [7,8,9,10,11,12]. In less than 10 years, we have seen progress in developing modified electrospinning methods, such as: Two-stream electrospinning; co-axial electrospinning; reactive electrospinning; and layer-by-layer electrospinning (LBL)—enhancing the end use spectrum [13,14,15,16]. Although we have produced nanofibres from more than 100 individual polymers [17], developing a green electrospinning process remains a challenge, largely because most polymers have poor solubility in water and there is a very limited supply of neutral polymers that are soluble in water—charged polyelectrolytes form the majority of water-soluble polymers. Since like charges repel each other, the viscosities of polyelectrolyte solutions where salt is absent are a good deal higher than those of neutral polymer solutions with similar polymer concentration. Almost all reported electrospun polyelectrolytes, whether neutral or salt, use toxic solvents in spinning and may need cross-linking agents to achieve chemically stable fibre mats [18,19,20,21]. The cytotoxicity of cross-linking agents and solvents has prevented extensive uses of fibre mats based on water-soluble polymers.

Chitosan (CHT) is a biodegradable polymer comprising of randomly distributed b-(1-4)-linked d-glucosamine (deacetylated) and *N*-acetyl-d-glucosamine (acetylated) units, which contribute to a cationic nature, as well as other properties. It is the cationic property that gives CHT the ability to penetrate mucous layers in biomedical applications, perform an antimicrobial function in food preservatives, and trap dyes and metals in waste water [22,23,24,25,26]. It is also being used in technical applications such as packaging and decontamination, because of its physico-chemical properties, such as hydrophobicity, thermal stability, and mechanical performances [27,28,29]. These properties, unique to CHT, combined with high porosity and surface area by its nanosize, render it important for a range of different functional systems. Nevertheless, a number of factors increase the difficulty in electrospinning CHT, such as: Poor solubility in most organic solvents; crystal formation; high molecular weight; polycationic characteristics in solution; H-bonding in the d-glucosamine unit; and a wide distribution of molecular weights [30]. Polyvinyl alcohol (PVA) can be added to reduce CHT viscosity. With a semi-crystalline molecular structure, PVA is a biodegradable and biocompatible synthetic polymer combining solubility in water with a nontoxic nature, rendering it widely usable in biomedical and biochemical applications. PVA is easy to spin, easy to coagulate, easy to orient, and easy to cross-link [31]. Attempts have been made to produce a high surface area nanofibre from a blend of CHT and PVA by electrospinning [32,33,34], but involved in the process were expensive and toxic chemicals including trifluoroacetic acid (TFA), 1,1,1,3,3,3-hexafluoro-2-propanol, dichloromethane, chloroform, and acetic acid. Any residues of these chemicals can have undesirable long-term environmental impacts and be a health hazard, so these nanofibres are not considered green. Therefore, the use of functional fibres of this sort is difficult to justify in applications demanding non-toxicity and environmental friendliness.

Many factors dictate electrospun nanofibres’ diameter: Solution parameters; process parameters; and ambient parameters [35]. Rigorous monitoring of these parameters is required to obtain the desired properties. The traditional approach has been to change only one parameter at a time, keeping all others fixed [36]. We have used the Taguchi experimental design approach to optimise our experiments [37,38].

Therefore, our broad aim in this work is to replace organic solvent electrospinning of water-insoluble polymers with water-based formulations i.e., green electrospinning. To that effect, water-soluble chitosan (W_S_CHT) was synthesised under mild and heterogeneous reaction conditions (H_2_O_2_-assisted) and prepared in an all ‘green’ approach to fabricate W_S_CHT/PVA-blended nanofibres using water as a non-toxic solvent for the very first time in literature. Another aim is to optimise the electrospinning process parameters to get a finer fibre diameter range using the Taguchi approach.

## 2. Experimental

### 2.1. Materials

High molecular weight chitosan (degree of deacetylation 65%), was purchased from (Sigma–Aldrich, Dorset, UK). PVA (M_w_: 115,000) was purchased from (BDH Ltd Poole, UK). The following chemicals were all obtained and used as reagents: Sodium hydroxide, hydrogen peroxide, hydrochloric acid, and ethanol. All the chemicals were used without further purification.

### 2.2. Synthesis of Water-Soluble Chitosan (W_S_CHT)

The following synthesis protocol was based on a previously reported method [39] with a number of modifications, as described further. CHT was completely dissolved in 1% (*v*/*v*) aqueous hydrochloric acid to a concentration of 1% (*w*/*v*), then 30% (*v*/*v*) H_2_O_2_ aqueous solution was added and the solution was then maintained in a thermostatic water bath at 65 °C for 40 min. The final solution was then filtered using Whatman filter paper to expel polluting influences, and to promote decontamination. The final product was acquired after dialysis using a Slide-a-Lyzer dialysis cassette (Pierce, MWCO 2000) (molecular weight cut-off) in deionised (DI) water for 12 h at room temperature. Then the pH of the solution was brought to 7 by adding few drops of 5 M NaOH solution, and precipitation was obtained by adding ethanol. This step was followed by another rinsing with hot water to ensure complete removal of NaOH. Then a solid was collected after drying the precipitate in a vacuum, which was then washed with ice cold water in order to remove the excess traces of acid and gently cast onto polystyrene dishes and dried at 25 °C to obtain pure W_S_CHT. Table 1 shows the properties of W_IS_CHT and W_S_CHT.

### 2.3. Preparation of W_S_CHT-PVA Nanofibre

**Scheme 1:**
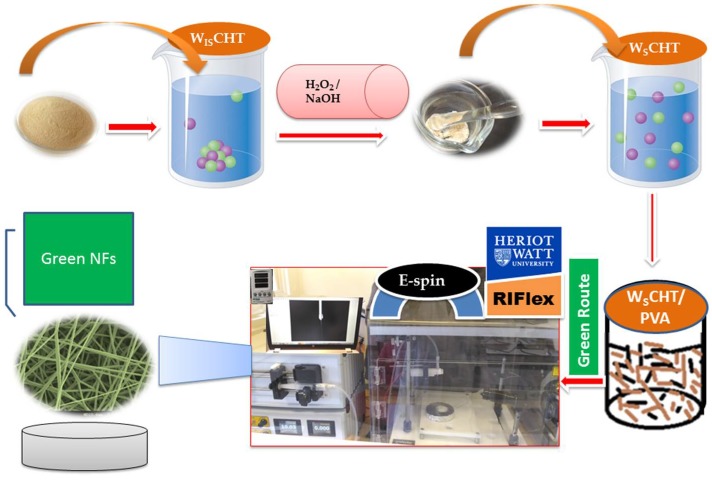
Schematic of synthesis of ‘green’ water-soluble chitosan/polyvinyl alcohol (W_S_CHT-PVA) nanofibre.

For preparing water-based electrospinning, solutions of W_S_CHT-PVA were made in various concentrations, as listed Table 2, by using water as a common solvent. Firstly, different W_S_CHT concentration solutions were prepared by dissolving the required amount of W_S_CHT powder in water (milli-Q grade) with gentle magnetic stirring for 56 h at room temperature to form a homogeneous solution. Different concentrations of PVA solutions were prepared by dissolving PVA in double-distilled water under magnetic stirring for 4 h at 75–80 °C, and then cooled to room temperature. Finally, the two polymer solutions were mixed at different weight ratios ultrasonically for 4 h to ensure a well-distribution of blended solutions before being subjected to the electrospinning process. The electrospinning experiment was carried out using an electrostatic spinning instrument (Research Institute for Flexible Materials, Heriot-Watt University, Edinburgh, UK), as shown in Scheme 1. The prepared blend solution was drawn into a 5 ml disposable plastic syringe equipped with a metallic needle (inner diameter gauge of 22). The flow rate was monitored by a syringe pump (TS2-60, Longer Precision Pump Co. Ltd., Baoding, China). A high voltage power supply was applied to the solution in order to generate electrostatic field between the grounded collector (copper collector plate measuring 15 cm × 15 cm) and the syringe needle tip. Aluminum foil covered the grounded collector plate to collect the electrospun nanofibres. Finally, the collected nanofibres were dried at 25 °C under vacuum for 24 h to remove the residual solvent. All experiments were conducted at room temperature with a relative humidity of less than 40%.

### 2.4. Taguchi Design of Experiment (DoE)

The work has been designed in a sequence of experimental steps to ensure that the collection of data satisfies statistical rules. This DoE and Taguchi-based DoE are simple methods of using orthogonal arrays to simulate a variety of experimental conditions. It is used to reduce experimental errors, process variation and for enhancing efficiency resulting in optimised process parameters and experimental reproducibility, whilst reducing time and cost of experimentation [37]. Work time and cost are reduced in the processes [39]. The method works through the conversion of target features to a signal to-noise (S/N) ratio, which allows the level of controlling parameters in contradiction to these parameters to be measured. S/N is the favourite signal ratio for random noise and assesses the quality of the experimental values. Three dissimilar functions were used as the target: ‘Larger is better’, ‘nominal is best’, and ‘smaller is better’. Using the S/N ratios allowed achievement of the optimum electrospinning condition. Then optimum electrospinning conditions were determined by the Taguchi optimisation method to validate the optimisation [40,41].

Table 3 shows the four parameters selected at each of the three levels: Larger, nominal, and smaller. Factors with different levels were combined in a manner matching the L_9_ type orthogonal array, Table 4. A ‘smaller is better’ characteristic formula determines the optimum combination of factors that would produce the smallest diameter electrospun nanofibres in accordance with Equation (1):

(1) $$\frac{S}{N}=-10\log\left(\frac{1}{n}\overset{n}{\underset{j=1}{\sum }}y_{i}^{2}\right)$$

where S/N is the signal-to-noise ratio, *n* is the number of observations, and *y* is the diameter of nanofibres measured. Software Minitab 17 was used for optimisation of the electrospinning process by the Taguchi approach.

### 2.5. Measurements and Characterisation

The viscosity of the solutions was measured using a viscometer (DV-II, Brookfield Co., Stoughton, MA, USA). The surface tension of the solutions was tested by a surface tensiometer (KRÜSS GmbH, Hamburg, Germany). The conductivity of the solutions was measured by a conductivity meter (HANNA HI8733, Leighton Buzzard, UK). All measurements were carried out at room temperature. The surface morphologies of the electrospun nanofibres were examined on a scanning electron microscopy (SEM; Hitachi, S-4300, Tokyo, Japan) at an accelerating voltage of 5 kV, and a working distance of 14 cm. Prior to the observation, the samples were sputter-coated with gold–palladium to keep away from charge gathering for 3 min in a Denton Desk II Sputter Coating Unit (Denton Vacuum, Moorestown, NJ, USA) for better conductivity during imaging. The diameters of nanofibres were measured by using an image analyser (Image J, National Institutes of Health, Bethesda, MD, USA).

## 3. Results and Discussion

### 3.1. Mechanism and Characterisation of W_S_CHT

Solubility has been considered a pivotal characteristic for CHT. CHT is insoluble in both water and most organic solvents, in spite of the fact that it is soluble in aqueous diluted acids. The poor solubility of chitosan turns into a significant restricting property in its usage, for example, in the case of biological applications of chitosan where many enzymes are performed at neutral pH media. The water-soluble CHT could be an important new modification that will enhance the biological and physiological properties of chitosan [42]. In addition, its high molecular weight is the principle cause for the low solubility of CHT in water. Therefore, it is intriguing to enhance the solvency of CHT by diminishing its molecular weight. Hydrogen peroxide turned out to be an effective tool to ideally degrade the crude CHT into water-soluble chitosan (W_S_CHT) in this work. The mechanism is due to the formation of reactive hydroxyl radicals by the disassociation of hydrogen peroxide. Hydroxyl radicals can attack the glycosidic linkages of chitosan and subsequently break the chain [43]. The overall reaction is shown in Equation (2) [44]:

H_2_O_2_ + R–NH_2_ + H^+^ → R–NH_3_^+^ + HOO^−^ + H^+^ (2)

The hydroperoxide anion (HOO^−^) is very unstable and readily decomposed to highly reactive hydroxyl radical (HO·) by Reactions (3) and (4) [45]:

HOO^−^ → OH^−^ + O^·^ (3)

H_2_O_2_ + HOO^−^ → HO^·^ + O_2_·^−^ +H_2_O (4)

The HO· reacts with a hydrogen atom from chitosan to form water, shown in Equation (5) [46]:

(CS)m–(CS)n + HO^·^ → (CS)m–(CS)n + H_2_O (5)

During this process, the R-NH_2_ generally connected with H^+^ to produce R-NH_3_^+^, which causes a decrease of H^+^ and an increase in pH. In addition, HOO^−^ is rapidly decomposed to HO^·^ which means that H_2_O_2_ is continually decomposed, as shown in Equation (1). These radicals undergo further reactions rapidly to form water-soluble oxidation products, i.e., chitosan, as mentioned in Equation (5).

### 3.2. Optical Micrograph

**Figure 1:**
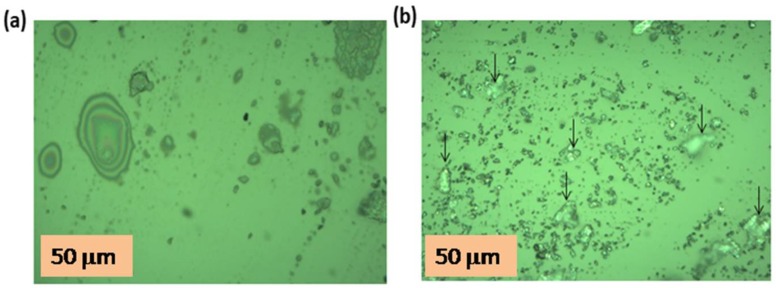
Optical micrographs of (**a**) water-insoluble chitosan (W_IS_CHT) and (**b**) water-soluble chitosan (W_S_CHT). The arrows in the image indicate the locations of W_S_CHT particles.

Figure 1a,b show the typical optical microstructures of W_IS_CHT and W_S_CHT. From Figure 1a, it can be seen that the microstructure of W_IS_CHT appears to show smooth surface, as contrasted with W_S_CHT (Figure 1b). Most of them were of unequal sizes and frequently an inadequate circular shape. This might be because of the inconsistency and the poor interfacial interaction [47]. Meanwhile, for the W_S_CHT sample in Figure 1b, white spots were observed on the surface (swelled extensively), and isolated into numerous little pieces with totally round shape structure, because of the internal intention during swelling. These white spots indicate W_S_CHT particles. Optical microscopy of all samples revealed good evidence of clear lamellar spacing throughout all samples, and similar results have also been reported by other researchers [48].

### 3.3. UV-Vis Spectra

**Figure 2:**
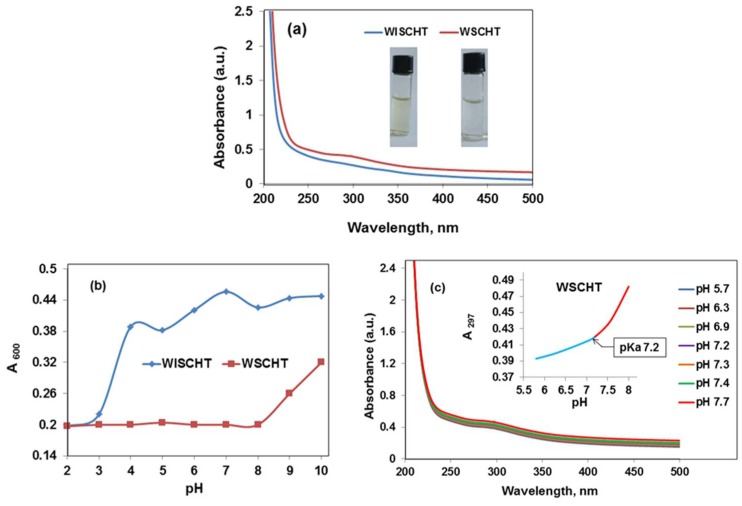
(**a**) UV-Vis spectra, (**b**) pH dependence solubility, and (**c**) pKa value.

UV-Vis spectroscopy is used to study covalent and non-covalent interactions between CHT and its solvents or dopants. Functional groups in organic molecules absorb light at characteristic wavelengths in the UV-Vis region, and this technique is used to identify the presence of those groups in samples. Ultrapure water provided the reference solution in obtaining the spectra of a CHT solution. W_S_CHT formation was confirmed by recording the absorbance of the colloidal suspension in the 200 to 500 nm UV range and by colour change observation. The colour of the solution became transparent, indicating that W_S_CHT had been synthesised. Figure 2a shows the UV spectra of both the original chitosan and the chitosan depolymerised by treatment with mild H_2_O_2_. Degraded chitosan exhibited a new absorption band in the range 280 to 300 nm due to a n–π* transition for the amino group and a π–π* transition for the carbonyl or carboxyl groups [49]. Each of the two peaks showed an increase in value with a decrease in the M_w_, and this is due to the formation of carbon-oxygen double bonds after the main chitosan chain bond had been broken and the hydrogen abstraction reaction had been followed by ring opening [50]. The original chitosan spectrum is also shown, with no peak in this wavelength range.

The pH-dependence of solubility was then examined, because the polymeric amphoteric compound’s behaviour in aqueous media appeared important for flocculent end users. Incident light scattering at 600 nm was used to indicate the solubility in water of W_IS_CHT and W_S_CHT solutions. W_S_CHT had no turbidity and was therefore water-soluble at pH values between 2 and 8 (Figure 2b, red line). W_IS_CHT, on the other hand, was insoluble at pH values above 3. UV spectra analysis showed W_S_CHT to have higher solubility than W_IS_CHT at acidic and neutral pH. Previous studies have shown solubility to be increased by a degree of chemical modification including acetylation and breaking of intra- and inter-hydrogen bonds of the chitosan. In the same way, there were hints of structural change in W_S_CHT. Linear raw chitosan polymers stack and form crystalline structures, but chemical modification takes away the polymers’ intrinsic crystalline character, as the polymers’ rigidity decreases and they become amorphous [51,52].

A molecule’s ionisation constant (pKa) is important as a physicochemical parameter influencing its solubility, lipophilicity, and protein binding, as well as its permeability. pKa therefore gives the pH at which ionisation reaches 50%. There are usually differences in the physicochemical properties of neutral and ionised forms of compounds: Water solubility and membrane permeability, for example, differ in this way, and they are important features of pharmaceuticals and environmental agents [53]. Compounds with pKa < 4 or >10 will be charged at physiological pH and their rate of diffusion across biological membranes (like in the blood–brain barrier) will be slower [54]. Figure 2c shows an increase in W_S_CHT’s pKa value after chemical modification to a level greater than the pKa of chitosan due to hydroxyl groups. This may cause an increase in W_S_CHT’s effective pKa value, and that in turn could be a factor in the increased solubility of W_S_CHT.

### 3.4. ^1^H NMR Analysis

**Figure 3:**
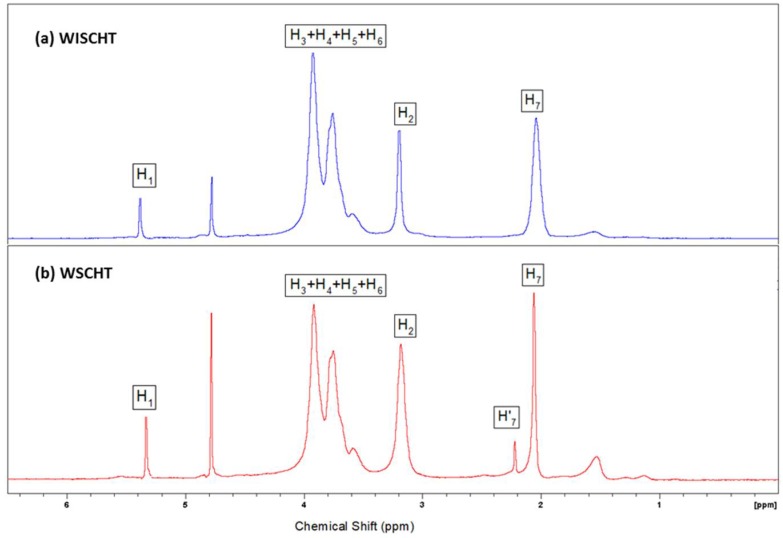
^1^H NMR spectra of (**a**) W_IS_CHT and (**b**) W_S_CHT.

To better understand the modified chitosan, we performed proton (^1^H) NMR spectroscopy of samples. It has been generally acknowledged as a basic instrument to give data on chemical environment and structure of materials that are not effortlessly noticeable by other non-ruinous high-determination spectral methods [55]. The ^1^H NMR spectrum of W_IS_CHT and W_S_CHT is shown in Figure 3a,b respectively. In case of W_S_CHT, it exhibited an absorption peak at 2.091 ppm (H-7), corresponding to the ring methane protons, because of the N-acetyl glucosamine units having survived the saponification chitin, a single at 3.20 ppm (H-2), four absorption peaks at 3.67–4.02 ppm (H-3, H-4, H-5, and H-6), and also a small peak at 5.34 ppm (H-1). Compared with the ^1^H NMR spectrum of W_IS_CHT, the shift of each 1H of W_S_CHT corresponds to the shift of chitosan, so the significant changes in characteristic shifts of W_S_CHT do not take place in comparison to W_IS_CHT in the spectra. In other words, the structure of W_S_CHT remains the same as that of the W_IS_CHT, which supports in the previous findings in literature [39].

### 3.5. Green Electrospinning

Green Electrospinning was proposed as a medium of fabricating nanofibres by electrospinning from aqueous concentrations, through the utilisation of nontoxic solvents (benign solvents) [21]. In addition, this technique helps to overcome the restrictions of too high polymer concentrations (solid content) of polymer solutions as aqueous suspensions for the electrospinning process [56]. Biodegradable polymers, for example, chitosan and Polyvinyl alcohol, have a variety of attributes that make them appealing material for the generation of nanofibres, including high/low molecular weights, biodegradability, and sourcing from renewable feedstocks [57]. Therefore, ‘green’ electrospinning of biodegradable W_S_CHT and PVA (W_S_CHT-PVA) from aqueous suspensions could be a promising way to deal with environmental issues of electrospinning with organic solvents. The morphology and performance of the electrospun fibre mats are being investigated, through the physical properties of the polymer solution and the parameters of the electrospinning process, as reported in this study.

### 3.6. Physical Properties of Solution

**Figure 4:**
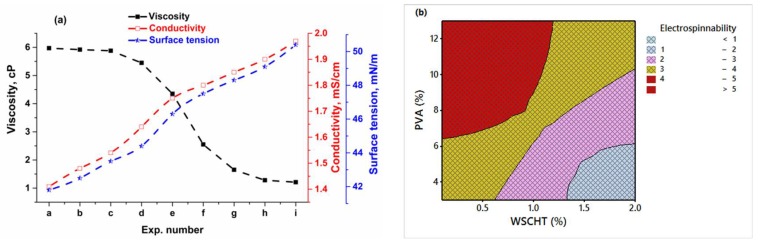
(**a**) Physical properties and (**b**) Contour plot the spinnabilities of W_S_CHT vs. Poly(vinyl alcohol) (PVA) solution.

The solution’s physical properties are known to play a significant role in electrospinning [58,59]. The solution properties affecting fibre diameter are: Viscosity η; conductivity σ; and surface tension γ [60,61]. We measured these properties, and the results are presented in Figure 4a. The W_S_CHT-PVA blend solutions’ viscosities decrease as W_S_CHT content increases. In electrospinning, tensile stresses affect the stability of the jet ejected at the Taylor’s cone [62], with the potential for a significant degree of elongational flow and a consequent stretching of the jet. The elongational flow characteristics can be found from the solution’s elasticity and viscosity. Jet stretching during electrospinning is found to be greater when W_S_CHT content is higher, and so an increase in the blend’s W_S_CHT content will lead to a reduction in the diameter of the fibres.

The solution’s electrical conductivity also influences fibre morphology. Electrical conductivity is related with electrical charges in the solution, and affects instability in electrospinning. Adding charge carriers, such as salts or conductive filler particles, influences the solution’s conductivity and may lead to two results with opposite effects on formation and diameter of fibres: An increase in the flow rate, leading to larger fibres; or an increase in net charge density which, by suppressing the Rayleigh instability and enhancing the whipping instability, leads to bead-free fibre formation and smaller diameter fibres [63]. The W_S_CHT-PVA blend conductivity is presented in Figure 4a: As it is seen, increases in W_S_CHT content were accompanied by increases in the blend’s conductivity. This is probably due to higher concentrations of free ions and other species likely to be ionised. This high conductivity can lead to finer fibres in agreement with [59].

Surface tension is a property in the liquid signifying that the liquid molecules are strongly cohesive, and that phases at the interface differ [64]. The W_S_CHT-PVA blend surface tension is presented in Figure 4a, which shows an increase in surface tension as W_S_CHT content increases. Surface tension of water is much higher than surface tension of most solvents, increasing the difficulties in electrospinning with water, as there is more likelihood of the jet breaking up into droplets and/or of beaded fibres being formed. The present study showed, to some extent, that increasing the amount of W_S_CHT in water solvent helps to increase the solution’s total surface tension, thanks to water’s relatively high surface tension. Increases in the solution’s surface tension significantly reduced the electrospun fibres’ diameters, while reducing surface tension in the spinning solution helps electrospinning to occur when the electric field is lower [65].

The contour plot in Figure 4b suggests that a stable spinning process with homogenous deposition of W_S_CHT-PVA nanofibres without beads from water is achieved, as depicted by the red area in Figure 4b. The formulations with W_S_CHT and PVA concentration are in the yellow and light purple regions of Figure 4b, although stable electrospinning inhomogeneous nanofibres and nanofibres with small amount of beads were obtained. The worse electrospinning process was found in the light blue area, where discontinuous electrospinning was observed.

### 3.7. Morphologies of W_S_CHT-PVA Nanofibres

**Figure 5:**
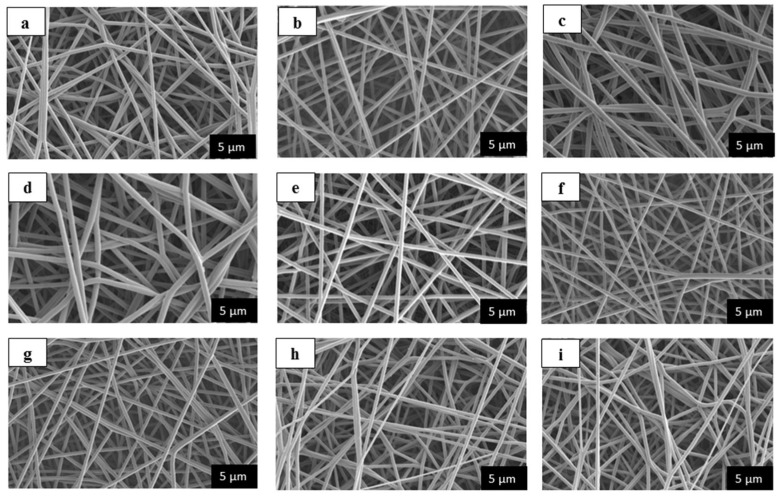
SEM images of W_S_CHT-PVA nanofibres (**a**–**i**), produced according to experiment design shown in Table 4.

**Figure 6:**
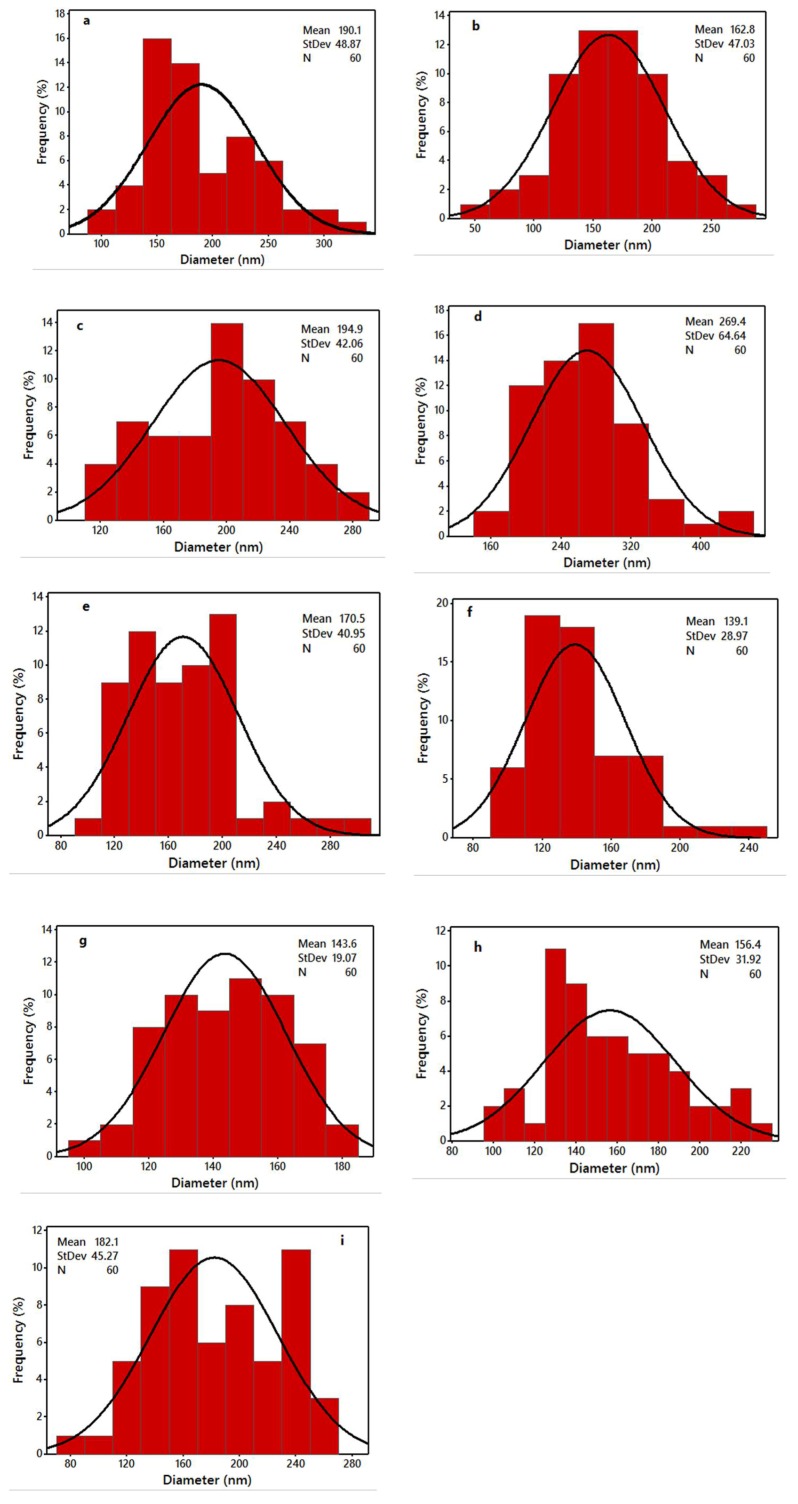
Diameter distribution histograms of W_S_CHT-PVA nanofibres (**a**–**i**), produced according to the experimental design shown in Table 4.

Figure 5 represents the surface morphologies of electrospun W_S_CHT-PVA nanofibres. When these were inspected by the SEM (Table 4), the morphology of electrospun nanofibres was found to be free of beads, smooth, and homogeneously distributed. Figure 6 shows the range of diameters (a range between 139.1 nm and 269.4 nm). Image J software was used to find the standard deviations of W_S_CHT-PVA green nanofibres. In the DoE study, experiment g gave the highest average diameter (269.4 nm), possibly explicable by the effect of introducing W_S_CHT in reducing the viscosity of the blended spinning solution. As the W_S_CHT content in the spinning solutions increased, the average diameter of green nanofibres is reduced. This concurs with the findings of [66]. The fibrous membranes’ structures showed greater uniformity, and there was a narrower distribution of nanofibre diameters. CHT can, in fact, be used to thicken electrospinning PVA solutions for more uniform fibres [67]. This is because adding W_S_CHT leads to a higher charge density on the surface of the jet, thanks to its ionic character. Overall, tension in the fibres is known to depend on the degree of self-repulsion from excess charges on the jet. Increasing the charge density on the jet therefore leads to a reduction in the blend fibers’ diameters [68]. On the other hand, further increases in W_S_CHT content (from 1.3% to 1.9%) hinder the electrospinning of W_S_CHT-PVA blend solution, because the Taylor cone is distorted, the spinning jet is unstable, and the nozzle clogs, stopping the spinning process and preventing fibre formation (Table 2).

Reports indicate that W_S_CHT’s rigid chemical structure, specific intra- and inter-hydrogen bond interactions, along with solution viscosity make it difficult to spin and poses difficulties in electrospinning of continuous fibres [69]. In an attempt to deal with these problems, we mixed WsCHT with PVA as a non-ionogenic partner suitable for preparing W_S_CHT-PVA blend nanofibres. This resulted in a more continuous electrospinning process.

### 3.8. Taguchi’s Method Optimisation

Taguchi experimental design is a more effective process evaluation and optimisation technique than traditional one-factor-at-a-time approaches [70,71]. Each product or process’s performance characteristic is assigned a nominal or target value and the aim is a reduction in variability around this value. Working conditions generated as optimal by the experimental study should give performance values in different operating environments, or at different times, that are either identical or very close to each other, and the optimisation criterion should be the controlling factor in minimising the performance value’s variability. The Taguchi method defines such optimisation criteria as performance statistics [72]. Each factor’s main effects are studied to give the optimisation criterion, and it is from these main effects that the factors’ general tendencies are deduced. Once it has been established that the desired result will be given by a low or a high value, the levels of the factors likely to yield the best results can be calculated. In the present study, ‘Minitab-17’ statistical software was used to calculate S/N ratio values by rearranging results according to a ‘smaller the better’ formula. The S/N ratio for this study should be the minimum value that gets the optimal electrospinning condition agreement with the Taguchi method.

### 3.9. Mean Effect Assessment 

**Figure 7:**
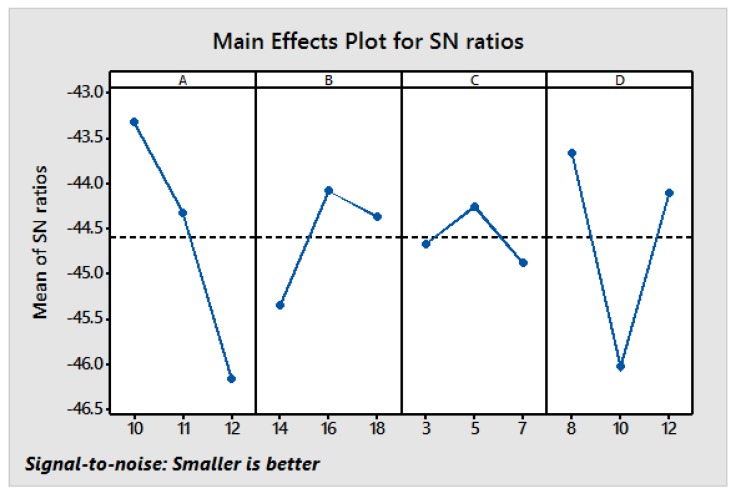
Main effects plot for signal-to-noise (S/N) ratios [Response: Diameter].

To assess the selected factors’ role in the sample’s average fibre diameter, it is necessary to calculate each factor’s main effect at each level. Figure 7a shows the mean effect of total solution concentration (%) (W_S_CHT + PVA), voltage, flow rate, and tip-to-distance on the average nanofibre diameters. As Figure 7a indicates, the average nanofibre diameter increases as the solution concentration increases. Justification for this observation would rest on a general assumption of consistency between solution viscosity and polymer concentration. During the electrospinning process, fibre formation depends on an optimum solution viscosity. Low solution viscosities lead to the formation of beads rather than nanofibres. In this study, increasing the percentage of W_S_CHT in the total concentration increases average fibre diameter due to increasing resistance. Applied voltage sharply decreases average fibre diameter. The literature also stresses the importance of voltage on fibre diameter. Very high voltage levels accelerate jet extension, causing more polymer solution to be drawn from the needle and larger diameter fibres to be formed [73]. On the other hand, strong electrical charge can stretch the polymer solution and decrease fibre diameter [74]. Sill and Recum have shown that increasing voltage beyond a critical value causes an initial reduction in fibre diameter, due to greater jet stretching followed, by increases in diameter [75]. Yördem et al. suggested that the voltage effect on fibre diameter may depend on the type of polymer-solvent system involved and the collection distance [76]. As Figure 7a shows, the flow rate increases to 0.7 mL h^−1^ with increased fibre diameter. There is, however, a limit to the increase in diameter which depends on feed rates and determines the rate at which the electrospinning jet is being carried away. Charge must increase as the feed rate rises, so that the solution stretching rises and offsets the increase in fibre diameter caused by increases in volume [77]. Figure 7a suggests a slight increase in average fibre diameter as the spinning distance increased, until it reached 10 cm, and thereafter any other increasing of the distance sharply reduces it. As the needle-tip-to-collector distance increases, the polymer solution jet is more able to bend and whip. Since bending and whipping of the jet are key factors in reducing the diameter of spun fibres in electrospinning [78], increases in collecting distance reduce nanofibre diameter.

The response table for the mean of means (diameter) and the mean of S/N ratio analysis are presented in Table 5, which helps to analyse the effect of control factors on the basis of delta statistics. The delta statistic for each factor is that factor’s highest figure after subtraction of its lowest average. The highest delta value belongs to the factor with the most influence on fibre diameter. The results reveal that solution concentration is found to have the strongest effect, with the highest delta on fibre diameter. Spinning distance is the second important factor, followed by voltage, and flow rate. Table 5 and Figure 7 show that the optimum levels for all varied factors to achieve the finest nanofibre are: Solution concentration of 10%; voltage of 16 kV; flow rate of 0.7 mL h^−1^; and distance of 8 cm.

**Figure 8:**
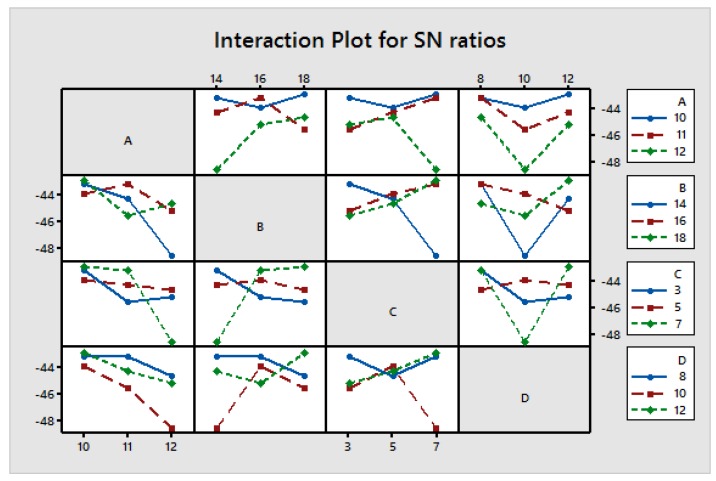
Interaction plot for S/N ratios [Response: Diameter].

Figure 8 displays a full interaction matrix plot for all variable electrospinning processing factors. An interaction effect between the chosen parameters can be studied by this plot. Interaction is said to exist if the relationship is shown by nonparallel lines, whilst parallel lines indicate that no relationship exists between those parameters [79]. In this interaction plot, it is observed that non-parallel lines exist between all parameters, which shows that the selections of these input parameters are vital to study the optimisation of the electrospinning process.

In order to investigate which process parameters significantly affect fibre diameter and to determine the percentage contribution of each operational variable, analysis of variance (ANOVA) was performed (Table 6). From Table 6, it is clearly identified that the solution concentration has the highest influence (49.31%) on diameter, followed by spinning distance (37.77%), voltage (10.54%), and flow rate (2.38%). Since there is no error in Taguchi L_9_ ($3^{4}$) orthogonal array at first, and the impact of each factor reaches a certain influence in the quality characteristic, there is no need to pool errors [80].

### 3.10. Confirmation Test

The confirmation experiment is a crucial step and is highly recommended by Taguchi to verify the experimental results [81]. The purpose of the confirmation test is to validate conclusions drawn during the analysis phase. Once the optimum level of the process parameters is found, the final step is to predict and verify the improvement of the performance characteristics using the optimum level of the process parameters. In this study, a confirmation experiment was conducted by utilising the levels of the optimal process parameters (A1B2C2D1) for fibre diameter, and the results are presented in Table 7. The increase of S/N ratio from the initial parameters to the level of optimal parameters is −1.14 dB for fibre diameter. From the confirmation tests, good agreement between the predicted performance and the actual performance is therefore evident.

**Figure 9:**
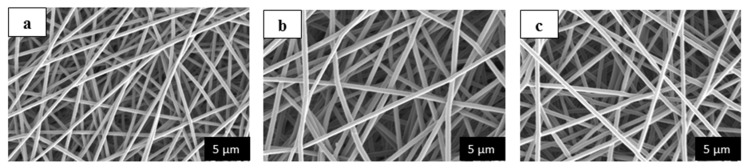
SEM images of the optimum condition experiment (**a**) and two different needle sizes: (**b**) 22 G and (**c**) 24 G under optimum condition.

**Figure 10:**
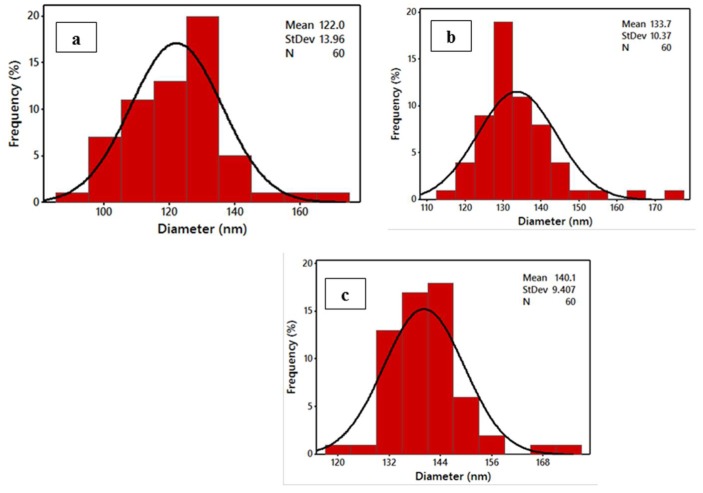
Diameter distribution histograms of the optimum condition experiment (**a**) and two different needle sizes: (**b**) 22 G and (**c**) 24 G under optimum condition.

Figure 9 shows typical SEM images of confirmation experiments using 22 G and 24 G needle sizes. The randomly oriented nanofibres had smooth surfaces without beads or any defects in all images. The mean diameter and range are 133.7 nm and 140.1 nm for the confirmation experiments of needle 22 G, and 24 G, respectively with a narrower distribution of diameters is shown in Figure 10. It is clear that needle size is a non-significant factor in nanofibre diameter of electrospun W_S_CHT-PVA. The diameters of these nanofibres for both needles sizes are compared closely with the best Taguchi candidate values, which obtained a fibre diameter of 120 nm.

## 4. Conclusions

In this study, W_S_CHT has been synthesised through heterogeneous based reaction and used for fabrication of nanofibres containing PVA as guest polymer (W_S_CHT/PVA), via a green electrospinning method with water as the solvent. First, W_S_CHT has been produced via H_2_O_2_ medium, and from optical micrographs it was shown that particles of W_S_CHT exhibited round shaped structure, as further confirmed by UV-Vis spectra analysis, and by the proton (^1^H) NMR spectra showing the corresponding shift of peaks. The physical properties of the W_S_CHT/PVA solution blend were obtained as reported, and morphological analysis using SEM has confirmed the uniformity of the electrospun fiber with standard distribution.

Furthermore, the process parameters were optimised using the Taguchi method, and conditions affecting the morphology of the produced nanofibres were established. From ANOVA, it is clearly identified that the solution concentration has the highest influence (49.31%) on diameter, followed by spinning distance (37.77%), voltage (10.54%), and flow rate (2.38%). In addition, the obtained optimum combination was validated by conducting a confirmatory experiment with improvement of S/N ratios, and proving the application of Taguchi’s method for electrospinning optimisation. This research shows how environmentally sustainable nano materials may be developed for many diverse end-uses e.g., biomedical science (drug carriers, antimalarial products, antiviral, etc.), entomology (to control mosquitoes and ticks) and for environmental applications.

## Figures and Tables

**Table 1:** Properties of raw and final product.

Sample	Viscosity cP	Conductivity mS/cm	Surface Tension mN/m	M_w_ (×10^−4^)	DD (%)	Yield (%)
W_IS_CHT	3.7	1.42	42	3.2	65	24
W_S_CHT	2.4	1.51	44	1.2	88	52

**Table 2:** Experimental condition in synthesis of nanofibre.

Sample Code	Polymer Blend (W_S_CHT + PVA) *w*/*v*%	Fibre Morphology	Diameter (nm)
WP1	0.1% + 12.9%	Semi-continuous fibre	284.2
WP2	0.3% + 11.7%	Continuous fibre	212.1
WP3	0.5% + 10.5%	Continuous fibre	187.1
WP4	0.7% + 9.3%	Continuous fibre	160.3
WP5	0.9% + 8.1%	Semi-continuous fibre	151.6
WP6	1.1% + 6.9%	Fibre with some beads	141.4
WP7	1.3% + 5.7%	Only droplets	-
WP8	1.5% + 4.5%	Not spinnable	-
WP9	1.7% + 3.3%	Not spinnable	-
WP10	1.9% + 2.1%	Not spinnable	-

**Table 3:** Selected factors and their levels used in Taguchi’s design.

Factors Level				
	Unit	1	2	3
A. Total solution concentration (SC)	%	10	11	12
B. Voltage (V)	kV	14	16	18
C. Flow rate (FR)	mL h^−1^	3	5	7
D. Distance (Dt)	cm	8	10	12

**Table 4:** L_9_ orthogonal array design of experiment (DoE).

Experiment No.	Factors				Diameter (nm)	S/N Ratio (dB)
	A	B	C	D		
a	1	1	1	1	144.4	−43.19
b	1	2	2	2	156.4	−43.88
c	1	3	3	3	139.1	−42.86
d	2	1	2	3	162.8	−44.23
e	2	2	3	1	143.6	−43.14
f	2	3	1	2	190.1	−45.57
g	3	1	3	2	269.4	−48.60
h	3	2	1	3	182.1	−45.20
i	3	3	2	1	170.5	−44.63
Optimum	1	2	2	1	122.0	−41.72

**Table 5:** Response table for means of signal-to-noise (S/N) ratios.

Level	Means of S/N Values			
	A	B	C	D
1	−43.31	−45.34	−44.66	−43.66
2	−44.32	−44.08	−44.25	−46.02
3	−46.15	−44.36	−44.87	−44.10
Delta	2.84	1.27	0.62	2.37
Rank	1	3	4	2

**Table 6:** Analysis of variance for S/N ratios.

Source	DF	SS	MS	P (%)
A	2	12.4003	6.2002	49.31
B	2	2.6508	1.3254	10.54
C	2	0.5989	0.2994	2.38
D	2	9.4998	4.7499	37.77
Residual Error	0			
Total	8	25.1497		

**Table 7:** Confirmation experiment.

Level	Initial Parameters	Prediction	Confirmation Experiment
	A_1_B_3_C_3_D_3_	A_1_B_2_C_2_D_1_	A_1_B_2_C_2_D_1_
Diameter	139.1	138.2	122.0
S/N	−42.86	−42.99	−41.72
Improvement of S/N ratio		−1.14	

## Abbreviation

DoE	Design of experiment
OA	Orthogonal array
ANOVA	Analysis of variance
DF	Degree of freedom
MS	Mean square
P (%)	Percentage contribution
SS	Sum of square
S/N ratio	Signal-to-noise ratio
R-Sq (R^2^)	R-squared statistic
W_IS_CHT	Water-insoluble chitosan
W_S_CHT	Water-soluble chitosan
